# Recent Advances in Machine Learning for Network Automation in the O-RAN

**DOI:** 10.3390/s23218792

**Published:** 2023-10-28

**Authors:** Mutasem Q. Hamdan, Haeyoung Lee, Dionysia Triantafyllopoulou, Rúben Borralho, Abdulkadir Kose, Esmaeil Amiri, David Mulvey, Wenjuan Yu, Rafik Zitouni, Riccardo Pozza, Bernie Hunt, Hamidreza Bagheri, Chuan Heng Foh, Fabien Heliot, Gaojie Chen, Pei Xiao, Ning Wang, Rahim Tafazolli

**Affiliations:** 1 Samsung Electronics R&D Institute, Staines TW18 4QE, UK; m.hamdan@samsung.com; 2 School of Physics, Engineering and Computer Science, University of Hertfordshire, Hatfield AL10 9AB, UK; 3 Professorship of Communications Engineering, Chemnitz University of Technology, D-09111 Chemnitz, Germany; dionysia.triantafyllopoulou@etit.tu-chemnitz.de; 4 5GIC & 6GIC, Institute of Communication System, University of Surrey, Guildford GU2 7XH, UK; r.a.borralho@surrey.ac.uk (R.B.); e.amiri@surrey.ac.uk (E.A.); d.mulvey@surrey.ac.uk (D.M.); r.zitouni@surrey.ac.uk (R.Z.); r.pozza@surrey.ac.uk (R.P.); b.hunt@surrey.ac.uk (B.H.); c.foh@surrey.ac.uk (C.H.F.); f.heliot@surrey.ac.uk (F.H.); gaojie.chen@surrey.ac.uk (G.C.); p.xiao@surrey.ac.uk (P.X.); n.wang@surrey.ac.uk (N.W.); r.tafazolli@surrey.ac.uk (R.T.); 5 Department of Computer Engineering, Abdullah Gul University, Kayseri 38080, Turkey; abdulkadir.kose@agu.edu.tr; 6 School of Computing and Communications, InfoLab21, Lancaster University, Lancaster LA1 4WA, UK; w.yu8@lancaster.ac.uk; 7 School of Science, Technology and Health, York St John University, York YO31 7EX, UK; h.bagheri@yorksj.ac.uk

**Keywords:** open radio access networks, machine learning, artificial intelligence

## Abstract

The evolution of network technologies has witnessed a paradigm shift toward open and intelligent networks, with the Open Radio Access Network (O-RAN) architecture emerging as a promising solution. O-RAN introduces disaggregation and virtualization, enabling network operators to deploy multi-vendor and interoperable solutions. However, managing and automating the complex O-RAN ecosystem presents numerous challenges. To address this, machine learning (ML) techniques have gained considerable attention in recent years, offering promising avenues for network automation in O-RAN. This paper presents a comprehensive survey of the current research efforts on network automation usingML in O-RAN.We begin by providing an overview of the O-RAN architecture and its key components, highlighting the need for automation. Subsequently, we delve into O-RAN support forML techniques. The survey then explores challenges in network automation usingML within the O-RAN environment, followed by the existing research studies discussing application of ML algorithms and frameworks for network automation in O-RAN. The survey further discusses the research opportunities by identifying important aspects whereML techniques can benefit.

## 1. Introduction

Open Radio Access Network (O-RAN) is a revolutionary concept in the field of wireless telecommunications that aims to transform traditional, proprietary Radio Access Networks (RAN) into open, intelligent, and interoperable networks. The O-RAN concept involves separating the hardware and software components of RANs and enabling interoperability and integration of solutions coming from different vendors. This open architecture is made possible through the use of open Application Programming Interfaces (APIs), standardized interfaces, and virtualization technologies, which allow RAN components to be disaggregated and easily swapped out or upgraded. Thus, O-RAN is expected to bring greater flexibility, innovation, cost-efficiency, design flexibility, operational adaptability, system functionality, deployment scalability, and function expandability to RANs, while also supporting the demands of next-generation networks and services such as 5G/6G, Internet of Things (IoT), and edge computing.

Over the past couple of decades, wireless communications have gone through several transformations to support massive connectivity and to meet the demands of modern real-time and mission-critical applications, as the target 5G and 6G Key Performance Indicators (KPIs) include ultra-high reliability, low latency, and high-throughput. However, despite of all advancements made in wireless systems, particularly in 5G, achieving all these goals remains challenging. The key problems confronting stakeholders in 5G and 6G systems include efficiently supporting wireless access across diverse frequency bands, dealing with heterogeneous technologies, addressing a wide variety of application requirements leading to complex protocol stacks, and managing the rising capital and operational expenditure (CapEx and OpEx) needed for infrastructure upgrades and maintenance [1]. These challenges include the need to design an independent, service-focused network architecture due to the diversity of Quality of Service (QoS) requirements, achieving network agility while ensuring backward compatibility with existing equipment and support for future upgrades, and guaranteeing network efficiency to avoid increased computational complexity and a heavy load on the backhaul network [2].

The motivation behind the development of the O-RAN concept lies on the fact that traditional RAN systems are proprietary, i.e., closed systems, limiting mobile network operators (MNO) to obtaining all the radio, hardware, and software systems from a single supplier when deploying a network at each region. Aside from the considerable impact on RAN deployment CapEx and OpEx, this implies the lack of openness and interoperability, which can hinder innovation and agility. Traditional RANs are monolithic systems, which are designed to operate as integrated products, seen by the operators as black-boxes. Traditionally, at its most basic level, the RAN architecture consists of a radio unit (RU) or remote radio unit (RRU), a baseband unit (BBU), antennas, and various software-based interfaces. As described above, this results in difficulty meeting the very strict and diverse KPIs of modern networks. Consequently, the consensus was that the mobile network should be more software driven, virtualized, flexible, and intelligent to provide all the KPI goals and address the aforementioned challenges.

The RAN evolution towards O-RAN started with disaggregation, defined by 3GPP in Release 15 [3], where the 5G NR RAN (more specifically gNB) functionalities split into three logical nodes: the Central Unit (CU), the Distributed Unit (DU), and the Radio Unit (RU). The CU handles gNB functions like transfer of user data, radio access management, positioning, mobility, and session management. A DU function is dependent on the functional split option, but mainly manages baseband processing functions across cell sites. The CU operation is controlled by the CU. The RU component is located near or integrated into the antenna unit where the radio signals are transmitted, received, amplified, and digitized. In traditional RAN configuration, sometimes called distributed RAN (D-RAN), BBU and RRH are co-located in the same place in the cell site, in which they are directly connected via Common Public Radio Interface (CPRI). Disaggregation option provides new levels of flexibility and efficiency at RAN level by enabling the network operators to decide where to locate each function and maximize performance. In 2009, centralized or cloud RAN (C-RAN) has emerged as an efficient solution, exploiting disaggregation to move the BBU functionalities to a centralized location, called BBU pool, while leaving the RRU and antenna on cell site. The principle design idea of C-RAN is to move some of RAN functionalities to the cloud infrastructure, the BBU pool could be implemented on a cloud platform [4]. The path towards O-RAN making mobile networks “more software driven, virtualized, flexible, intelligent and energy efficient”, as well as “cost-efficient and reliable” [2], is paved through the use of Network Function Virtualization (NFV) concepts. The O-RAN concept is supported by several standard bodies such as the O-RAN Alliance [5], the Third Generation Partnership Project (3GPP) [6], the European Telecommunications Standards Institute (ETSI) [7], the Next Generation Mobile Networks (NGMN) [8], and the Optical Internetworking Forum (OIF) [9], to ensure interoperability and interconnection between O-RAN components from different vendors.

As O-RAN environments are inherently complex, characterized by heterogeneity and dynamism, with various hardware and software components from different vendors working together, Machine Learning (ML) is expected to be an invaluable tool. The effectiveness and efficiency of O-RAN architectures are intricately tied to the integration of ML capabilities. ML techniques offer a plethora of advantages within O-RAN architectures. ML algorithms excel at real-time analysis of extensive network data, including KPIs, end-user behaviors, and network traffic patterns, all in real-time. This analytical strength empowers the prompt identification of trends, anomalies, and performance issues, facilitating network optimization and predictive maintenance [10]. Moreover, ML plays a vital role in resource allocation by intelligently assigning radio resources, optimizing resource utilization, and enhancing QoS [11]. Additionally, ML algorithms facilitate data-driven decision-making in O-RAN management and orchestration functions, such as automated network configuration, dynamic spectrum allocation, and intelligent traffic steering. This streamlined approach reduces network management complexity and empowers MNOs to optimize network performance. Overall, ML is a very important tool for O-RAN as it allows for the provision of insights, efficient resource allocation, and automated management capabilities.

In this paper, we embark on a comprehensive exploration of the current landscape concerning ML applications in O-RAN. Our objective is to identify and analyze the prevailing challenges that remain unresolved, preventing the full harnessing of ML’s potential for enhancing O-RANs. While there have been several O-RAN survey papers in the literature [1,4,12,13,14,15,16,17,18,19], our survey paper stands out as it concentrates on the applications and potential of ML within the O-RAN context, which has not been extensively addressed in prior surveys. Table 1 provides a summary of the topics covered in relevant surveys, along with their contributions, in order to provide a clear comparison with our work. It can be noted that most of the existing surveys aim to provide a detailed tutorial on RAN evolution, O-RAN architecture, and components and use cases. They all have different focuses compared to our paper. For example, ref. [16] specifically focuses on the security and privacy risks associated with Open RAN architecture, which complements our survey. Meanwhile, ref. [17] centers its attention on the Non-Terrestrial Network (NTN), offering an architectural solution for an O-RAN-based NTN system. In addition, although [18,19] both acknowledge the prevailing challenges and prospective research directions in this field, ref. [18] primarily examines the existing O-RAN specifications, while [19] focuses on how Explainable AI (XAI) can contribute to O-RAN networks. Among the existing survey papers, refs. [14,15] have a similar focus on ML in O-RAN. Ref. [14] mainly provides a tutorial on how intelligent applications can improve the efficiency of O-RAN and the future opportunities in O-RAN, while [15] looks into how deep learning solutions can be integrated to the O-RAN architecture, as well as case studies. However, ref. [15] primarily focuses on deep learning techniques rather than general ML techniques. Moreover, while [14,15] discuss open problems and future research directions, they primarily look at these from the perspective of O-RAN, rather than looking at the specific challenges and opportunities associated with ML-empowered O-RAN. Different from the existing surveys, in this article, we dive deep into the ML integration in O-RAN, covering recent research works that study the application of ML in O-RAN, discussing emerging issues and research opportunities that shall be addressed to fulfill its design commitment. We believe this survey marks a significant milestone as the first comprehensive endeavor aimed at summarizing recent studies and providing crucial technical guidance to researchers interested in ML-enabled O-RAN. Within this paper, we also present research opportunities across diverse areas encompassing data collection and analysis, as well as the development, deployment, maintenance, and operation of ML within the O-RAN domain.

**Figure 1:**
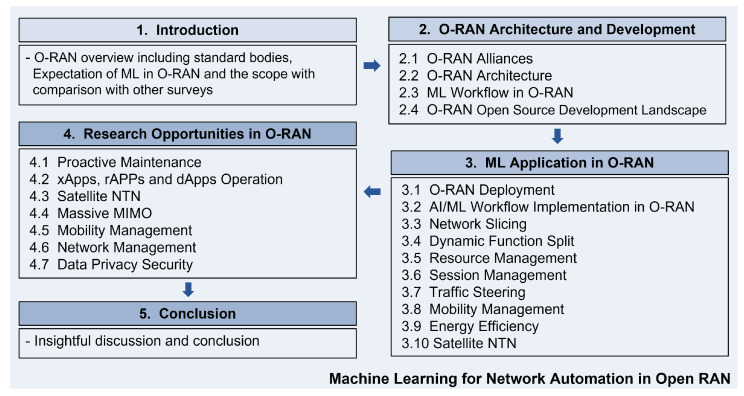
The overall structure of this paper.

The structure of the remaining sections in this paper is as follows. Section 2 provides an overview of the O-RAN architecture and its development, highlighting its design principles that support network automation. In Section 3, we survey the existing ML applications in the context of O-RAN. Section 4 focuses on the potential of O-RAN and discusses the research opportunities for applying ML techniques to enhance various aspects of mobile network operation within the O-RAN framework. Finally, Section 5 concludes the survey and discussion presented in this paper. We illustrate the organization of this paper in Figure 1.

## 2. O-RAN Architecture and Development

O-RAN architecture is a virtualized, software-driven, and open radio access network (RAN) architecture that enables the integration of hardware and software components from multiple vendors. It is designed to be modular, scalable, and flexible, with standardized interfaces that enable interoperability between different RAN components. The O-RAN architecture consists of multiple functional components that can be separated and managed independently. Some of the key components in O-RAN are Radio Unit (RU), Distributed Unit (DU), Central Unit (CU), RAN Intelligent Controller (RIC), and Service Management and Orchestration (SMO). In the following, we shall first describe the O-RAN alliance, architecture and softwarization developments, as well as their role in ML for O-RAN network automation.

### 2.1. O-RAN Alliance

The O-RAN Alliance is a global industry consortium that was founded in 2018 to drive the development and adoption of open and intelligent Radio Access Networks (RAN). The alliance is made up of more than 200 member organizations, including mobile network operators, network equipment vendors, and software companies.

The goal of the O-RAN Alliance is to create a more open, interoperable, and cost-effective RAN ecosystem that can accelerate the deployment of 5G networks and support new use cases and services. To achieve this goal, the O-RAN Alliance focuses on three main areas:

- Standardization: the O-RAN Alliance works to develop and promote open standards for RAN interfaces and APIs, enabling multi-vendor interoperability and reducing network deployment costs;
- Software: the O-RAN Alliance develops and promotes open software for RANs, including software-defined radio (SDR), virtualized RAN (vRAN), and open APIs for software integration;
- Testing and integration: the O-RAN Alliance provides specifications, conformance testing, and integration guidelines to ensure that O-RAN solutions can be easily integrated into existing network environments and can interoperate with other vendors’ solutions.

Through its work in these areas, the O-RAN Alliance is helping to create a more open, flexible, and efficient RAN ecosystem that can meet the demands of 5G networks and support new use cases and services, such as industrial IoT, autonomous vehicles, and smart cities.

### 2.2. O-RAN Architecture

The O-RAN architecture is a set of open interfaces and protocols designed to enable multi-vendor interoperability and support a wide range of use cases and services in RAN. The architecture defines a modular and disaggregated approach to building RAN systems, where different functional components can be developed and deployed independently by a variety of vendors. Such an approach intends to increase innovation, reduce costs, and enable faster deployment of new services and features.

**Figure 2:**
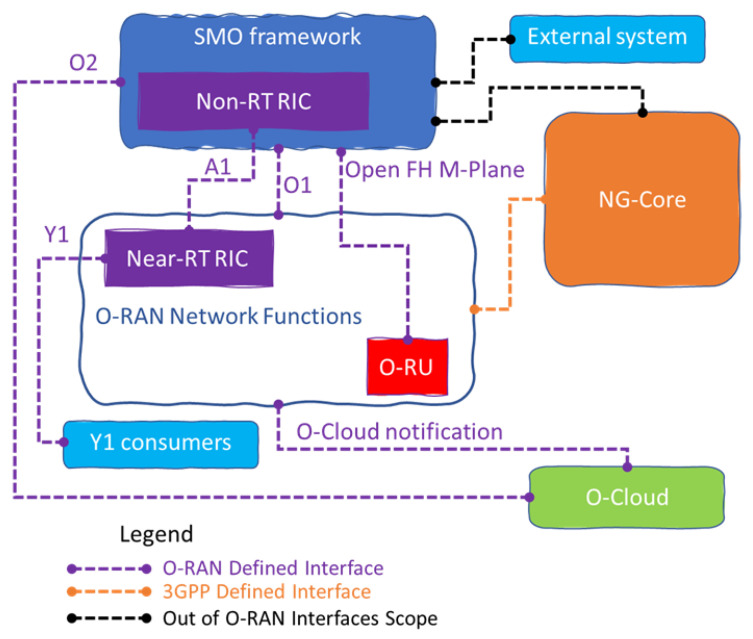
High-level architecture of O-RAN showing internal, 3GPP, and external system interfaces [20].

The O-RAN architecture specifies main components and interfaces connecting the components. The interfaces allow the Service Management and Orchestration (SMO) framework to connect with O-RAN network functions and O-Cloud. Figure 2 illustrates the high level O-RAN architecture, which can be viewed as Virtualized Network Functions (VNFs) placed above the O-Cloud and/or Physical Network Functions (PNFs). The A1 Interface between the Non-RT RIC in the SMO and the Near-RT RIC used for RAN Optimization, O1 Interface between the SMO and the O-RAN Network Functions used for Fault, Configuration, Accounting, Performance, Security (FCAPS) support and in the hybrid model. The Open Fronthaul M-plane interface between SMO and O-RU supports FCAPS too. While the O2 Interface between the SMO and the O-Cloud provides platform resources and workload management, the O-Cloud Notification interface allows event consumers such as an O-CU implemented on O-Cloud to subscribe to events or status. Moreover, the Y1 interface permits the Y1 consumers to subscribe or request the RAN analytics information delivered by Near-RT RIC. Where the Y1 consumer stands for an entity or more, within or outside of the public land mobile network (PLMN) trust domain that ingests analytics information services after mutual authentication and authorization by subscribing to or requesting the RAN analytics information via the Y1 service interface. There are three main control loops that run simultaneously in O-RAN, depending on the use cases, which are real-time (RT), which is limited to a maximum of 10 ms execution time $t_{exe}$; Near-RT, with $10\leq t_{exe}\leq 1000$ ms; and None-RT, which can take $1000\leq t_{exe}$ ms. Multi-vendor Slices use case targets enabling functions that belong to different vendors; there are many possible configurations to deploy the Multi-vendor slicing, all of which share that one O-RU is connected to one or more O-DUs. The advantages of such a use-case include a higher flexibility and rapid deployment of services to market by network operators, sharing RAN equipment among operators, optimizing CAPEX and OPEX among their existing assets, and future investments. In addition, reducing the supply chain risk; for example, if an existing vendor supplies a certain pair of vO-DU and vO-CU functions and if, for business reasons or even political situations, it has to withdraw from a certain market, then the operator can outsource and deploy alternative vO-DU and vO-CU that support multi-vendor slicing functions. The O-RAN specification work has been covered by eleven technical Work Groups (WG) to covers all the O-RAN Architecture parts, each WG has been supervised by the O-RAN alliance technical steering committee. Below is a brief overview of each WG:

- WG1: Use Cases and Overall Architecture. This WG is responsible for defining the overall architecture of Open RAN and the use cases that it will support;
- WG2: Non-Real-Time RAN Intelligent Controller and A1 Interface. This WG is responsible for defining the specifications for the Non-Real-Time RIC (Non-RT-RIC) and the A1 interface. The Non-RT-RIC is a centralized controller that manages the non-real-time aspects of the RAN. The A1 interface is the interface between the Non-RT-RIC and the radio units;
- WG3: Near-Real-Time RIC and E2 Interface. This WG is responsible for defining the specifications for the Near-Real-Time RIC (nRT-RIC) and the E2 interface. The nRT RIC is a centralized controller that manages the near-real-time aspects of the RAN. The E2 interface is the interface between the nRT RIC and the radio units;
- WG4: Open Fronthaul Interfaces. This WG is responsible for defining the specifications for the open fronthaul interfaces. The fronthaul is the interface between the baseband unit and the radio units;
- WG5: Open F1/W1/E1/X2/Xn Interfaces. This WG is responsible for defining the specifications for the open F1/W1/E1/X2/Xn interfaces. These interfaces are used to communicate between different parts of the RAN;
- WG6: Cloudification and Orchestration. This WG is responsible for defining the specifications for cloudification and orchestration of the RAN. Cloudification is the process of moving the RAN to the cloud. Orchestration is the process of managing the RAN;
- WG7: White-box Hardware. This WG is responsible for defining the specifications for white-box hardware for the RAN. White-box hardware is hardware that is not proprietary to a specific vendor;
- WG8: Stack Reference Design. This WG is responsible for defining the stack reference design for the RAN. The stack reference design is a model of the RAN that can be used to develop and test different RAN implementations;
- WG9: Open X-haul Transport. This WG is responsible for defining the specifications for open X-haul transport for the RAN. X-haul transport is the transport of data between the baseband units and the radio units;
- WG10: OAM. This WG is responsible for defining the specifications for operation, administration, and maintenance (OAM) for the RAN. OAM is the process of monitoring and managing the RAN;
- WG11: Security. This WG is responsible for defining the specifications for security for the RAN. Security is a critical part of the RAN, and this WG is responsible for ensuring that the RAN is secure.

**Figure 3:**
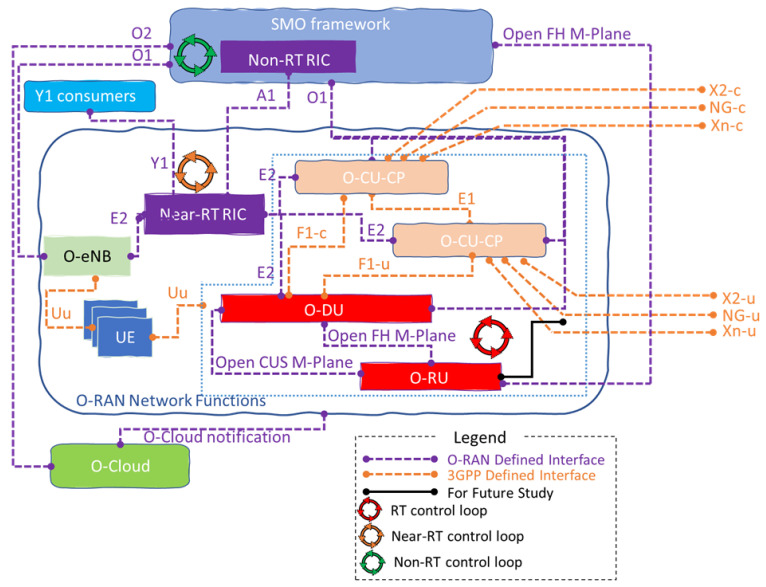
Logical architecture of O-RAN, its associated interfaces, and the three control loops.

Figure 3 provides further detail of the O-RAN architecture. As can be seen, O-RAN consists of O-RU, O-DU, O-CU, Near-RT RIC, Non-RT RIC, and SMO. The Uu interface between UE and O-RAN components inside the green dashed area, as well as the UE and O-eNB, denote all the O-RAN functions required to support the Uu interface NR. On the other hand, the O-eNB terminates the Uu interface for LTE. The 3GPP defined and maintained interfaces and is considered part of the O-RAN architecture includes the E1, F1-c, F1-u, NG-c, NG-u, X2-c, X2-u, Xn-c, and Xn-u, as depicted in Figure 3 [20]. In the following, we shall elaborate these O-RAN components.

#### 2.2.1. O-RU

**Figure 4:**
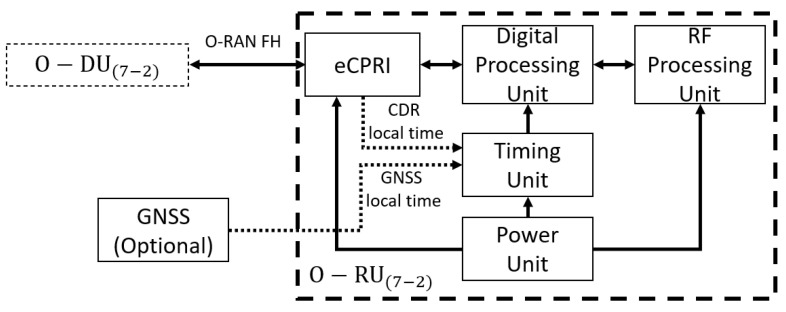
O-RU high-level architecture showing the main hardware components, and internal and external interfaces.

**Figure 5:**
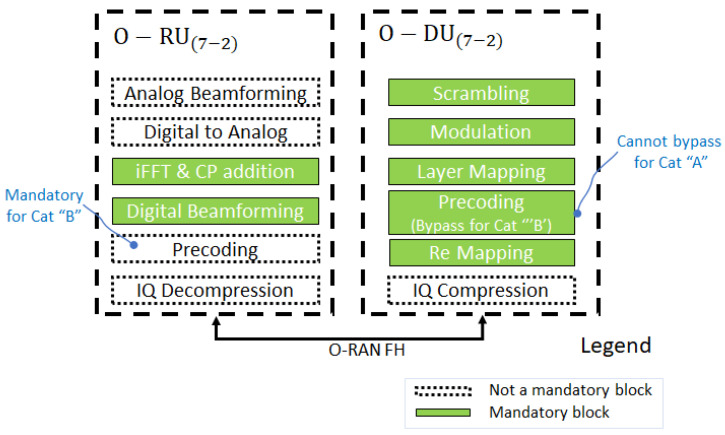
The O-DU${}_{\left(7-2\right)}$/O-RU${}_{\left(7-2\right)}$ split point option showing requirements for Category A and Category B for the O-RU.

The major O-RU hardware and software components in Figure 4 highlight the internal and external interfaces that are required. The O-RU terminates the O-RAN Fronthaul (FH) interface, known as Lower Layer Split, as well as Low-PHY functions of the radio interface towards the UE. This is a physical node. The O-RU terminates the O-RAN Fronthaul M-Plane interface towards the O-DU and SMO. The O-RU termination of the O1 interface towards the SMO is under study under the O-RAN Operations and Maintenance Architecture. A single split point, known as “7−2x”, but which allows a variation, with the precoding function located either “above” the interface in the O-DU or “below” the interface in the O-RU. For the most part, the interface is not affected by this decision, but there are some impacts, namely to provide the necessary information to the O-RU to execute the precoding operation. O-RU${}_{\left(7-2\right)}$ within which the precoding is not done (therefore of lower complexity) are called “Category A” O-RUs, while O-RU${}_{\left(7-2\right)}$ within which the precoding is done are called “Category B” O-RUs, as in Figure 5.

#### 2.2.2. O-DU

The O-DU is designed as a white box that performs the O-DU functions, such as upper L1 and lower L2 functions. The hardware includes a motherboard that contains a processing unit, memory, the internal I/O interfaces, and external connection ports. There are two split options for the O-DU, which are O-DU${}_{\left(6\right)}$ and O-DU${}_{\left(7-2\right)}$. WG4 considers the O-DU${}_{\left(7-2\right)}$ functional split option due to the two competing interests. The first is to keep an O-RU as simple as possible, because size, weight, and power draw are the primary deciding considerations, and the more complex an O-RU, the larger, heavier, and more power-hungry the O-RU tends to be. The second is to have the interface at a higher level, which tends to reduce the interface throughput relative to a lower-level interface. However, the O-RU tends to be the more complex with higher levels of interface.

The fronthaul and backhaul interface are used to carry the traffic between O-RU${}_{\left(7-2\right)}$, $FHM_{\left(7-2\right)}$, $FHGW_{(7-2)\to 8}$ and O-DU${}_{\left(7-2\right)}$, as well as O-CU and O-DU${}_{\left(7-2\right)}$. The O-DU${}_{\left(7-2\right)}$ design may also provide an interface for hardware accelerator option design. The other hardware functional components include synchronization and timing, the storage for software, hardware and system debugging interfaces, and board management controller, just to name a few; the O-DU${}_{\left(7-2\right)}$ designer will make decision based on the specific needs of the implementation. Note that the O-DU${}_{\left(7-2\right)}$ hardware reference design is also feasible for O-CU and integrated O-CU/O-DU${}_{\left(7-2\right)}$.

#### 2.2.3. O-CU

The O-CU is another white box hardware that performs the O-CU function of upper L2 and L3. The O-CU hardware motherboard contains a processing unit, memory, the internal I/O interfaces, and external connection ports. The midhaul (MH) is used to carry the traffic between O-CU and O-DU${}_{\left(7-2\right)}$, and the backhaul (BH) interface is for carrying the traffic between the O-CU and core network. Other hardware functional components, such as the storage for software, hardware and system debugging interfaces, board management controller, and more, are based on the specific needs of the implementation. The hardware of the O-CU is similar to the O-DU${}_{\left(7-2\right)}$. However, the hardware accelerator is mandatory to offload computationally intensive functions and to optimize the performance under varying traffic and loading conditions.

#### 2.2.4. Near-RT RIC

**Figure 6:**
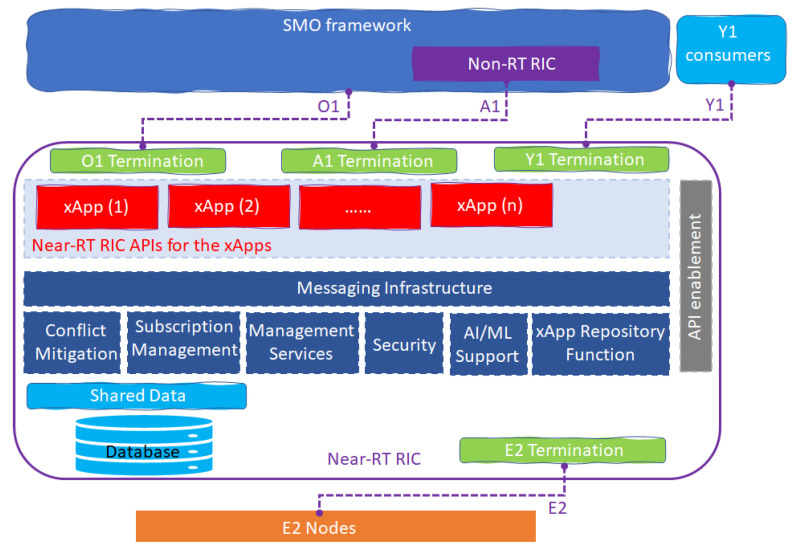
Near-RT RIC functional architecture including the Near-RT RIC API component for the xApps.

The Near-RT RIC is a logical function that can control and optimization RAN elements and resources in near real time by collecting detailed data from the O-RAN logical components and provide actions over the E2 interface. In addition, the A1 Interface enables Non-RT RIC to drive the policy guidance of the Near-RT RIC applications/functions and support AI/ML. Near-RT RIC hosts many functions in Figure 6, which include the following:

- Database and related Shared Data Layer (SDL) services: to exchange information between RAN and UE to support specific use cases;
- xApp subscription management: to manage subscriptions from different xApps and provides unified data distribution to xApps;
- Conflict mitigation: to resolve potentially overlapping or conflicting requests from multiple xApps;
- Messaging infrastructure: to allow message interaction within the Near-RT RIC functions;
- Security, which provides the security scheme for xApps;
- Management services: to manage fault, configuration and performance as a service producer to SMO;
- Logging service: to provide tracing and metrics collection which capture, monitor, and collect the status of Near-RT RIC internals and transfer to external systems for further evaluation if needed;
- Interface termination: to provide interfacing to other O-RAN components;
- Functions hosted by xApps: to allow services to be executed at Near-RT RIC;
- API-Enabled function: to support capabilities related to Near-RT RIC API operations such as API repository/registry, authentication, discovery, generic event subscription, etc.;
- AI/ML support: to feature data pipelining, training, and performance monitoring for xApps;
- xApp Repository function: to manage selection of xApps for A1 message routing, based on the A1 policy types and operator policies, and Access control of A1-EI types for xApps based on operator policies.

#### 2.2.5. SMO and Non-RT RIC

**Figure 7:**
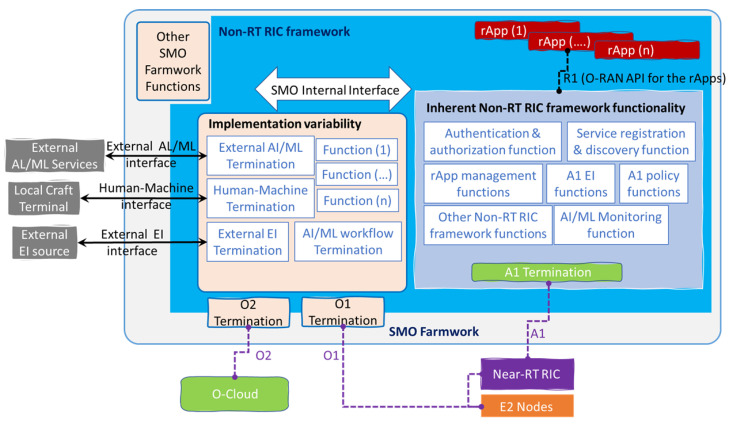
Non-RT RIC functional architecture, showing the R1 and external interfaces [21].

**Figure 8:**
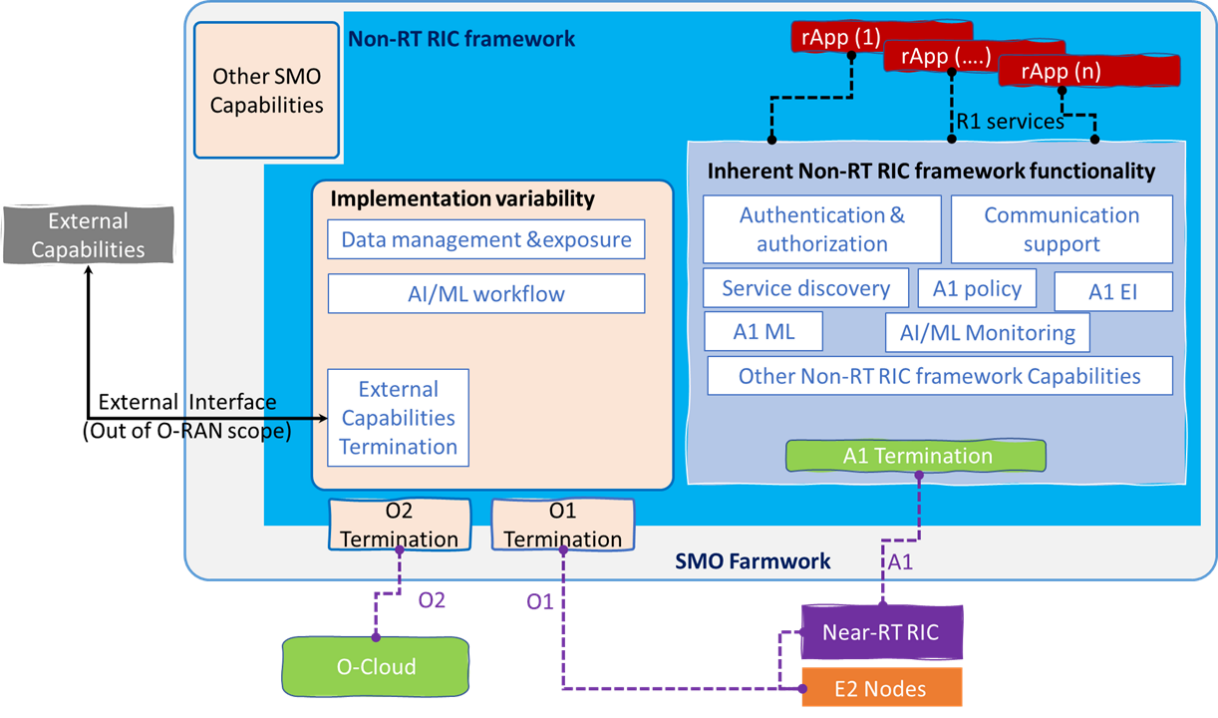
Non-RT RIC architecture service-based view, in which the services are exposed to rApps via the R1 [21].

The telecom industry widely considers the service-based architectural in network implementation to give flexibility and future-proof solutions. In addition, the choice of components that produce and/or consume certain services to the deployment allows multi-vendor interoperability through the definition of standardized services and service interfaces. It is important to have the perspective of two Non-RT RIC architecture views; the first one is the “Functional” view in Figure 7, showing the internal SMO framework and the three categorical components: rApps, Non-RT RIC framework and the open APIs for the rApps, while the “Service-based” view allows wide range of flexibility for deployment and is future-proof, the main principles for this architecture illustrated in Figure 8 are modularity, extensibility, functional abstraction, discoverability, composability, reusability, and loose coupling.

#### 2.2.6. O-RAN Interfaces

The O-RAN architecture is designed to promote interoperability, multi-vendor support, and innovation in the RAN. Here are the main interfaces specified in the O-RAN architecture:

- Open Front Haul (OFH) interface: this interface connects the O-RU to the O-DU and carries the digitized baseband signal and control information between the two units;
- Fronthaul interface: This interface connects the O-RU to the O-CU in the O-RAN Cloud RAN architecture. The interface carries the digitized baseband signal and control information between the O-RU and the O-CU;
- E1 interface: This interface connects the O-DU to the O-CU and carries control and management information between the two units;
- E2 interface: This interface connects the O-CU to the O-CU-CP and carries control and management information between the two units;
- A1 interface: This interface connects the O-RAN Controller to the O-RAN Element Management System (EMS) and provides management and monitoring capabilities for the O-RAN network;
- O1 interface: This interface connects the O-RAN SMO to the O-RAN Controller and provides service orchestration and management capabilities;
- O2 interface: This interface connects the O-RAN Controller to the O-RAN Radio Resource Management (RRM) and provides resource management and optimization capabilities for the O-RAN network;
- O3 interface: This interface connects the O-RAN Controller to the O-RAN Network Management (NM) and provides network management and monitoring capabilities.

#### 2.2.7. Interface with 3GPP

3GPP interfaces are also used in O-RAN to provide message exchanges between O-RAN components following 3GPP signaling specifications. They are summarized as follows:

- X2 interface: The X2 interface is used to exchange control information and user data between different eNodeBs (eNBs) in a 4G/LTE network. It is also used to facilitate inter-cell handovers and load balancing. O-RAN uses the same X2 interface for communication between O-RAN radio units and between the O-RAN radio units and 3GPP core network;
- S1 interface: The S1 interface is used to exchange control and user plane information between the eNodeB and the 4G/LTE core network. This interface is responsible for mobility management, session management, and connection management. O-RAN uses the same S1 interface for communication between the O-RAN radio unit and the 3GPP core network;
- F1 interface: The F1 interface is a new interface introduced by O-RAN that connects the O-RAN radio unit to the O-RAN distributed unit. This interface carries the radio frequency (RF) signals and also supports the exchange of control and management information. The F1 interface is similar to the W1 interface used in 3GPP’s split architecture;
- E2 interface: The E2 interface is used in the 5G RAN to exchange control and management information between different network functions, including the radio access network function (RANF), central unit (CU), and distributed unit (DU). The O-RAN Alliance has developed an E2 interface specification that is compatible with the 3GPP E2 interface.

### 2.3. ML Workflow in O-RAN

ML workflows in O-RAN involve a series of steps that enable the development, deployment, and optimization of ML models for network operations. The workflow consists of several stages of processing. We shall elaborate the processes involved in ML workflow in the following [22]:

- Data collection: The first step in the ML workflow is to collect and preprocess the data. This involves identifying the relevant data sources, collecting the data, and preparing it for analysis. This step is crucial as the quality of the ML model depends on the quality of the data used to train it;
- Data exploration and analysis: In this step, the collected data are explored to gain insights and identify patterns. This involves data visualization, statistical analysis, and other data exploration techniques to understand the underlying structure of the data. This step is important for selecting appropriate ML algorithms and for identifying relevant features that can be used to train the models;
- Model development: In this step, ML algorithms are selected and trained using the data. This involves selecting the appropriate algorithms, feature engineering, and tuning the model hyper-parameters. Once the model is trained, it is evaluated and validated to ensure that it is accurate and reliable;
- Model deployment: In this step, the trained model is deployed into the O-RAN environment. This involves integrating the model into the network operations environment and deploying it in a way that allows it to access the relevant data and provide real-time predictions or recommendations. This step also involves monitoring the performance of the deployed model to ensure that it is performing as expected;
- Model optimization: Once the model is deployed, it needs to be optimized to improve its performance. This involves monitoring the performance of the model in real-time, identifying areas for improvement, and updating the model as necessary. This step is crucial for ensuring that the ML models continue to provide accurate and reliable predictions and recommendations over time;
- Model maintenance: The final step in the ML workflow is model maintenance. This involves maintaining the ML model, updating it as necessary, and ensuring that it remains aligned with the evolving needs of the O-RAN network operations environment.

Overall, the ML workflow in O-RAN involves a series of steps that enable the development, deployment, and optimization of ML models for network operations. By leveraging the power of ML, network operators can improve network performance, reduce energy consumption, and provide a better user experience.

### 2.4. O-RAN Open Source Development Landscape

During the past few years, open source platforms for cellular networks have been developed to move away from proprietary hardware and mitigate technological barriers [23,24,25,26]. The main open source projects implementing O-RAN specifications are OpenAirInterface (OAI) [24], srsRAN [25] and O-RAN Software Community (O-RAN SC) [23]. These three communities are sharing the source code of different modules with different states of progress. For example, the OAI has implemented the CU/DU split by supporting Software Defined Radio (SDR) USRP devices [27] for RU and on-the-shelf UEs. The testbed Colosseum [28] was built to provide remote access to OAI resources configured with 256 SDRs. It is a large-scale wireless testbed with a massive channel emulator, which enables the design, development, and testing of solutions at scale in various deployments and channel conditions. The testbed is open to the research community and can be used for experimental research with different applications. On the other hand, srsRAN has developed full stacks of UE and gNB with a simple setup compared to the OAI platform. The O-RAN SC has published partly their industrial solutions of the O-DU with Medium Access Control (MAC) and Radio Link Control (RLC) protocols. In parallel, the Software-Defined Radio Access Network (SD-RAN) paradigm enables RAN programmability and introduces new APIs for control extending platforms like OAI and srsRAN to support control/data plane separation [29]. The objective of SD-RAN is to focus on the L2 protocols: Radio Resource Control (RRC), RLC, MAC and Packet Data Convergence Protocol (PDCP) protocols. FlexRAN [30] is an example of SD-RAN platforms promising flexibility by supporting dynamic control functions and robustness by handling network applications with critical real-time requirements. Other new SD-RAN controllers, such as the 5G-EmPower [31], deal with other challenges like heterogeneity of mobile RANs or RAN slicing with NexRAN [32]. These open source platforms and APIs represent key first steps toward the availability of a fully open-source O-RAN solution.

These platforms allow the research community to experiment with new methods and replace the simulation hypothesis. The research results would be with significant impact. Furthermore, without the shift of the softwarization of the RAN functions as close as possible to the antenna, the ML algorithms cannot be applied efficiently for the reconfiguration of the network. In addition, the 3GPP specifications are upgraded frequently with new interfaces and protocols to improve the global network performances and handle new applications. For example, the 3GPP release 17 introduces new NTN and satellite communications and the new Multicast/Broadcast Session (MBS) in the definition of protocols’ functions. If the RAN is closed with a full hardware implementation, the solution would not be maintained quickly and the research community would not have access to new challenges. However, the flexibility of the software radio would allow the designer and the researcher to quickly maintain the O-RAN solutions as well as design, prototype, demonstrate, and analyze the O-RAN functions in the real-world settings.

There remain challenges in the implementation and testing of open source O-RAN solutions. Limited use cases, missing functionalities in the current implementation, as well as the affordability of hardware devices are some obstacles to make further progress in O-RAN implementation. For example, at the time of writing, O-RAN SC [23] shares only the O-DU without the complete implementation of the CU. This O-DU handles the registration of one UE with one distributed unit and without the possibility to test it with an SDR. The srsRAN [25] and O-RAN SC [23] support the release 15 of the 3GPP specifications, and they do not support the side link V2X communications use case. The OAI [24] uses USRP X300, which is a capital outlay. Therefore, research and development efforts, such as developing a specific SD-RAN for existing RIC implementation (for example, FlexRIC [33]), or supporting new 3GPP specifications (for example, splitting of the CU into CU-UP and CU-CP [34]), are open for both research and industrial communities to contribute.

## 3. ML Application in O-RAN

One key advantage of the Open RAN architecture is its ability to separate intelligent controls from the core network. This architecture gives flexibility for RAN to incorporate intelligence toward network control. With this architecture, ML can be easily integrated into Open RAN, not only to automate and optimize network operations, but also to improve network efficiency, introducing new use cases and services that are traditionally challenging to implement in the RAN.

While being developed, the concept of Open RAN has already sparked many research works in investigating the potential of Open RAN and its performance benefits. Notably, substantial research works focusing on applying ML algorithms in Open RAN have appeared in the literature. Improvements, such as optimizing radio resource management and network slicing, automating component deployment for efficient use of computing and communication resources, or improving energy efficiency, are some research activities receiving attention. The structure of this section is summarized in Table 2, and the content in each subsection is summarized in Table 3, Table 4, Table 5, Table 6, Table 7, Table 8, Table 9, Table 10, Table 11 and Table 12.

### 3.1. O-RAN Deployment

The deployment of an Open RAN poses challenges in terms of computing and network resources. Integrating hardware and software components from multiple vendors can lead to issues with resource allocation, such as conflicts over computing power, storage, and bandwidth. Interoperability challenges can also lead to inefficiencies in resource utilization, as different components may have different requirements and capabilities. In addition, security concerns related to access control, data protection, and privacy can require additional computing and network resources to address. Overall, the deployment of O-RAN requires careful planning and management of computing and network resources to ensure optimal performance and security.

In [15], the authors investigate how the deep-learning mechanisms could be deployed in an O-RAN architecture, via its hierarchical RIC modules. In particular, its O-RAN placement within the Near-RT RIC, O-CU, O-DU, and O-RU modules, as well as the associated functional blocks and O-RAN interfaces, is discussed. The authors describe the general procedure to implement automated DL models in O-RAN to achieve stable performance of these models by introducing ML system operations (MLOps) concept in O-RAN. They then go deeper and explore two case studies for DL deployment in O-RAN, which are classified as supervised and/or deep reinforcement learning (DRL).

For supervised learning, the authors identify two approaches to deployment: a centralized approach and a federated learning (FL) distributed architecture. The authors propose that for either approach, in order to enhance RAN performance and reduce operational cost, RICs would integrate embedded ML capability. To achieve this, local models (i.e., “xAPPs”) would run in the Near-RT RIC and global model parameters would be generated by the Non-RT RIC. In the centralized case, the data would be held in the Non-RT RIC, but for FL, the xApps could be built in the Near-RT RIC using O-RU level data, and just the local parameters transmitted to the Non-RT RIC for aggregation, leaving the data to be held locally. In either approach, the Non-RT RIC would then send out the global parameters to the xApps to update their models, which then operate in real-time using data obtained from the O-RUs via the O1 interface.

In the case of reinforcement learning (RL), the authors focus the deployment strategy on the actions of the RL algorithm intelligent agent(s), which should be deployed near the Near-RT RIC to improve the performance of xApps. This agent then uses the E2 interface to communicate with the O-DU and O-CU-C/U modules to take periodic actions to update the policy of resource allocation and scheduling in the O-DU’s MAC layer.

Additionally, the agent connects to the O-RU through the O1 interface to receive the obtained reward based on user experience quality and new state of the system expressed by the total number of allocated resource blocks and users’ density. By using inputs and rewards, the ML model can be trained to make data-driven decision more accurately.

The authors then discuss options for control of the training and deployment process for ML systems. The first option is a fully manual process with review by network staff before deployment of an updated run-time system. The authors envisage that this will become inadequate, however, in cases where data profiles vary with time, requiring frequent retraining. They propose an automated pipeline process, triggered by various predefined criteria, to retrain, validate, and deploy the updated ML systems. Pipeline metadata are retained in case a roll-back to a previous model is required, and to assist in debugging. The performance of RAN, which is based on manually created and deployed ML models, may degrade due to the dynamics of the radio access environment, or even the data profiles of the environment; as a result, the authors propose a general procedure to implement automated DL models in O-RAN to achieve the stable performance of such O-RAN models, called MLOps. Furthermore, the Non-RT-RIC module can monitor the Near-RT-RIC performance for implementing the ML models using the A1 interface to pass the information to enable ML automation. In addition, the article shows that the O-RAN architecture supports the design of machine-learning-based schemes to provide optimization for the Automatic Neighbor Relation (ANR) function of a Self Organizing Network (SON), which allows gNodeB handovers process improvement and provides an example article [56], where both Acumos Framework and Open Network Automation Platform (ONAP) were used to create the ML models that the O-RAN RIC module can execute, monitor, and manage the model design workflow with.

Finally, the authors identify a number of open problems in the deployment of DL systems in O-RAN. A critical issue is security, where the large number of new interfaces and the potential lack of trust between the components results in a significant number of new threats. The authors also identify issues with the integration of network slicing, self-organizing network functions and edge computing entities, the use of online DL systems, scalability, and challenges with energy management.

In order to overcome the challenges with the integration of network slicing capabilities within the O-RAN environment, the authors of [35] tried to optimize the admission and placement of O-RAN slices using DRL. The authors emphasize that while previous works considered the placement of slices to optimize processing and bandwidth resources, they have not considered admission control or the long-term impact of admitting a slice. The authors propose an optimization model using a joint RL approach to intelligently admit and place network slices in the available resources of different load scenarios. The proposed solution is compared with two baseline methods, a greedy heuristic and a DRL-based solution (RMAX) [57], under two load scenarios, low and high load. The results show that the proposed solution outperforms the baseline methods in terms of revenue, cost, and total profit for both scenarios, and therefore maximizes the long-term profit of infrastructure providers by considering revenue factors of slices and the idle cost of servers to deploy them.

**Table 3:** Summary of current efforts on ML applications for O-RAN deployment.

Year	Ref	Contribution
2022	[15]	Proposed a centralized/federated learning approach for the deployment of deep learning mechanisms in the O-RAN architecture, while the integration of RL mechanisms is associated with the Near-RT RIC, O-CU, O-DU, and O-RU modules. The E2 and O1 interfaces are explored for RL-based resource allocation and scheduling.
2022	[35]	Proposed a joint DRL-based solution using PPO to solve an optimization problem aiming to intelligently admit and place network slices in the O-RAN environment considering the available resources and different network loads.

### 3.2. AI/ML Workflow Implementation in O-RAN

The O-RAN specification addresses the overall architecture and solution for AI/ML Workflow-related requirements in [22] for the O-RAN use cases. These requirements allow automating AI scaling, where Data, Model Training, and Model Evaluation pipelines are key points to make AI faster, easier to deploy, and able to scale to larger and more complex problems.

In [36], the authors adopt the importance of ML training pipeline automation to propose ML pipeline automation techniques to apply the MLOps level 1 (ML pipeline automation) to the RIC platform, where they use Kubeflow for supporting the end-to-end lifecycle of the model management and propose the training pipeline automation to the RIC Platform to conduct the online training process. However, the authors use the KFServing inference service to deploy Kubeflow’s trained model. The new ML xApp type structure removed the RMR/gRPC adapter and replace it by using a Shared Data Layer (SDL) and RIC Message Router (RMR) libraries directly from the ML xApp. The authors show that the round trip time of the inference request between assisted xApp and ML xApp reduced significantly when the number of requests is more than 300. To find how an RL model application performs under the proposed RIC AI/ML workflow, the authors trained it to solve the resource allocation in the DU optimization problem using PPO to show the improvement in user throughput.

**Table 4:** Existing studies on ML applications for AI/ML implementation in O-RAN.

Year	Ref	Contribution
2021	[22]	Provides an initial O-RAN standard the terminology, workflow, and requirements, related to AI/ML model training, distribution and deployment.
2021	[36]	Proposed ML pipeline automation technique to manage ML training in O-RAN RIC.

### 3.3. Network Slicing

Network slicing in O-RAN refers to the ability to partition a physical network into multiple virtual networks, each tailored to specific use cases or applications. Each network slice has its own set of resources, including bandwidth, processing power, and storage, which can be dynamically allocated and managed according to the needs of the specific use case. O-RAN network slicing enables operators to offer customized services to their customers, such as low-latency communication for industrial applications or high-bandwidth streaming services for consumers. It also allows multiple services to be delivered over the same physical infrastructure, maximizing resource utilization and improving efficiency. The O-RAN architecture provides a framework for implementing network slicing, with the RAN and the CN working together to manage and allocate resources. The RAN is responsible for managing the radio access resources, while the CN is responsible for managing the core network resources. Together, they can allocate and manage resources across the network slices as required.

The synergies between O-RAN and network slicing, as well as SON and MEC technologies, were explored in [58], where the O-RAN platform was proposed as a common denominator for the integration of those technologies via proper modifications and extensions of its present architecture. It has been shown that an O-RAN-centric approach is beneficial, and such integration solves some of the issues not well-addressed by the current O-RAN implementation. Also, due to the integration, some components of the contributory technologies can be removed or reused.

ML algorithms can be used to automate the process of creating, modifying, and deleting network slices based on changing network conditions. By combining predictive analytics, real-time monitoring, optimization, and intelligent resource allocation, ML algorithms can create a closed-loop feedback system that can automatically adjust network slices to meet changing network conditions. The main point is to enable the third party to develop better ML algorithms. Particularly for network slicing, we can capture data for training purposes so as to improve the efficiency of network slicing. Based on real-time monitoring, ML algorithms can identify changes in network conditions. By monitoring network performance, ML algorithms can detect changes in network traffic, demand, and capacity, and can modify network slices in response. Then, ML algorithms can be trained to learn how to optimize network slicing based on changing network conditions. By considering factors such as network traffic, user demands, and network capacity, machine learning algorithms can adjust the size and characteristics of network slices to improve network performance and reduce energy consumption. With the network slicing, ML algorithms can then optimize the allocation of network resources to different network slices based on changing network conditions. By learning from historical data and network performance metrics, machine learning algorithms can adjust the allocation of network resources to different slices in real-time, and can optimize resource utilization to improve network performance and reduce energy consumption.

In particular, ML can be of help to network slicing by (i) traffic forecasting; (ii) admission control; and (iii) resource allocation [37]. They reflect three key network slicing building blocks that, together, aim at ensuring network slicing service level agreements (SLAs) are fulfilled. The traffic forecasting block allows us to predict the evolution of traffic load and resource usage for slices over future time instants. The outcome of the traffic forecasting solution can be fed into the slice admission control solution and slice resource allocation solution to enable better decisions (e.g., maximize system resource utilization). The admission control decides upon the slices to be served over future time instants, based on different criteria, e.g., resource availability, resource efficiency, operator revenue, etc. It can also be built on the outcome of the traffic forecasting for refining admission decisions in an anticipatory manner. Once a slice/user is admitted, the resource allocation block assigns the resources to each slice/user by avoiding over-provisioning and under-provisioning of the resources and ensuring the SLAs are respected.

O-RAN is a promising RAN architecture that inherits all the necessary features, such as intelligence, open and standard interfaces, and closed control loops, to facilitate resource management in a network shared among verticals. In [38], AI techniques are used to perform predictions of future SLA violations and perform corrective actions in advance. Specifically, a recurrent neural network model is utilized to predict the amount of resources required over each slice, given the volume of traffic it carries. E2E O-RAN setup has been used for evaluation of the intelligent closed control loop and resource provisioning scheme, for network slicing and control of the radio and cloud resources of slices, respectively.

In O-RAN, distinct network slices must be dynamically controlled to avoid SLA variation caused by rapid changes in the environment. A novel framework is introduced in [39] to manage network slices through provisioned resources intelligently. Due to diverse heterogeneous environments, the intelligent machine learning approaches require sufficient exploration to handle the harsh situations in a wireless network and accelerate convergence. To tackle this issue, a solution based on evolutionary-based DRL (EDRL) is proposed to accelerate and optimize the slice management learning process in the RIC modules.

An elastic O-RAN slicing problem is addressed in [40] for industrial monitoring and control in the industrial internet of things (IIoT) networks. This work aims to reduce the age of information (AoI) penalty of fresh information updates from different IIoT devices while considering the energy consumption of the IIoT devices. A matching game for solving the IIoT association problem is introduced and an actor-critic-based DRL model applied for O-RAN slicing-based resource allocation.

**Table 5:** Summary of ML studies for network slicing in O-RAN.

Year	Ref	Contribution
2022	[37]	Introduced the application of ML to network slicing; discussed some open challenges and potential solutions.
2022	[38]	Provided an intelligent closed-loop SLA assurance scheme for O-RAN slicing. A real-world dataset of a large operator is used to train a learning solution for optimizing resource utilization in the proposed closed-loop service automation process.
2022	[39]	Developed a novel O-RAN slicing framework over an evolutionary-based DRL approach to manage network slices dynamically in the rapid changing environment.
2022	[40]	Addressed the elastic O-RAN slicing problem for industrial monitoring and control in IIoT and introduced a matching game for solving the IIoT association problem, and then applied an actor-critic-based deep reinforcement learning model for O-RAN slicing-based resource allocation.

### 3.4. Dynamic Function Split

Dynamic function splitting is an essential technique for enhancing the efficiency and flexibility of O-RAN. It involves breaking down a network node or application’s functions into smaller and more modular components, which can be distributed across different computing resources. By leveraging ML, O-RAN can benefit from real-time intelligence and decision-making capabilities. ML algorithms can analyze network traffic patterns and resource usage to predict future demand and allocate computing resources accordingly, leading to better resource utilization, reduced latency, and improved overall network performance. Additionally, ML can optimize the selection of computing resources for specific functions based on factors like location, processing power, and energy efficiency, resulting in better dynamic function splitting and better service delivery to users.

In [41], the authors provide a RL-based approach to the problem of optimizing dynamic function splitting in O-RAN compliant disaggregated and virtualized RANs. Their paper addresses the specific scenario of function splitting between a CU and one or more DUs in a fully virtualized environment, as opposed to splitting between virtual and physical resources. They adopt a multi-agent RL approach using either Q-Learning or State Action Reward State Action (SARSA); a similar but slightly different alternative to Q-Learning. Optimization takes into account the traffic type (e.g., eMBB or URLCC) and also energy efficiency. It is assumed that the CU and each of the DUs run on separate physical environments, each with their own renewable energy source backed up by a battery-based energy storage facility, with a grid connection as a reserve input. Optimization is designed to minimize operational expenditure (Opex), including maximizing the usage of the renewable energy source, taking into account the energy remaining in the battery and also the variation in grid electricity prices during the day. Optimization takes place over a 48-h period and comparative Opex results are presented using solar radiation data from Stockholm, Cairo, Jakarta, and Istanbul, combined with broad traffic level assumptions.

In [42], a novel and efficient energy-efficient RAN disaggregation and virtualization method tailored for O-RAN is presented. This method effectively tackles challenges related to dynamic traffic conditions. By formulating the energy consumption as a multi-objective optimization problem, the authors integrate the Advantage Actor-Critic (A2C) algorithm with a sequence-to-sequence model to effectively address the sequential nature of RAN disaggregation and capture long-term dependencies. The results demonstrate the effectiveness of the proposed solution to reduce energy consumption for dynamic Virtual VNF splitting over traditional approaches like D-RAN and C-RAN.

In [43], the authors propose a novel DRL-based algorithm to jointly solve the optimal placement of network functions between the CU, DU, and user RU in an O-RAN architecture. In the meantime, the proposed algorithm aims to minimize the end-to-end delay and deployment cost, while considering constraints such as processing capacity and link bandwidth. The proposed method evaluates the impact of user mobility on the proposed DQN-based joint user association and CU-DU placement scheme (DJRCD) using the SUMO traffic simulation tool. The algorithm is tested using a mixed highway-urban region in the north of the Greater Toronto Area in Canada, and it is found to be superior to existing methods, achieving a reduction of up to 30% and 40% in end-to-end delay and deployment cost, respectively.

**Table 6:** Summary of current efforts on ML applications for dynamic function splitting in O-RAN.

Year	Ref	Contribution
2021	[41]	Applied RL to dynamically perform function split decisions for DUs and CU in a virtualized O-RAN architecture.
2023	[42]	Applied DRL to propose an energy-efficient RAN disaggregation and virtualization approach across edge sites, including DUs, and a cloud site, including CUs.
2022	[43]	Proposed a DRL-based algorithm to jointly solve the optimal placement of network functions between the CU, DU, and user RU in an O-RAN architecture.
2023	[59]	Proposed a DRL-based algorithm for a multi-objective optimization to minimize computational costs and the overhead associated with periodically reconfiguring dynamic VNFs splitting.

The dynamic nature of O-RAN environments often necessitates VNF reconfigurations during operation, resulting in additional overhead costs and traffic instability. To tackle this challenge, the work in [59] introduced a multi-objective optimization approach aimed at simultaneously minimizing VNF computational expenses and the periodic reconfiguration overhead. This solution relies on constrained combinatorial optimization with deep reinforcement learning, where an agent works to minimize a penalized cost function derived from the proposed optimization problem. The evaluation of this solution demonstrates substantial improvements, including a remarkable 76% reduction in VNF reconfiguration overhead, accompanied by a modest increase of up to 23% in computational costs. Furthermore, when compared to the most resilient O-RAN system, Centralized RAN (C-RAN), which does not necessitate VNF reconfigurations, this solution delivers savings of up to 76% in bandwidth while revealing a 27% overprovisioning of CPU resources.

### 3.5. Resource Management

In [44], the authors compare the performance of on-policy and off-policy DRL methods. The former is based on Proximal Policy Optimization (PPO) and the latter on a Sample Efficient Actor-Critic with Experience Replay (ACER). This process was conducted under an O-RAN setup. The O-RAN architecture is a suitable technology for DRL implementation, since it includes mechanisms that enable AI for more efficient network management and orchestration. In particular, both Near-RT and Non-RT RICs are designed for hosting AI workflows, namely DRL models. Since the objective is to optimize the resource allocation of a real-time surveillance video application, the types of services were classified as latency-sensitive and latency-tolerant. In this direction, two slices are established and managed by an O-RAN cross-slice resource orchestrator hosted by the SMO. One slice serves video surveillance cameras to transmit real-time videos via O-RAN to the 5G Core Network (CN) and then to a Control Center (CC) for real-time monitoring, and a slice serving the latency-tolerant users. The performance of the DRL on-policy mechanism was shown to provide better overall results namely in terms of implementation simplicity, performance stability, good trade-off between users latency and energy consumption, and faster convergence.

In [45], the authors consider the problem in which the eMBB and URLLC services compete for limited and insufficient computing resources, and the operator must balance the allocation of these resources to users of both services in multiple O-RUs/shared O-Cloud while maximizing fairness. The problem is initially modeled as an Integer Linear Programming (ILP) problem. However, given the high complexity of solving the NP-Hard ILP problem, a policy gradient-based RL algorithm aiming to solve a Markov Decision Process (MDP) is used instead. The latter is expected to perform similarly to the ILP solver, and both approaches are compared. Simulation results showed that the RL agent performed close to the optimal results of the ILP solver not deviating from the ILP by more than 6%, while being fairer at the same time.

The authors of [46] proposed an RL-based framework to manage traffic flows while taking advantage of the O-RAN ecosystem. The framework receives periodic reports from the O-RAN DU about the network status and dynamically adapts the per-flow resource allocation for which each traffic flow can compete, and identifies the corresponding modulation and coding scheme (MCS) that best fits the traffic flow KPIs and the channel quality. The RL-based dynamic resource controller solution that leverages a policy differential semi-gradient State-Action-Reward-State-Action (SARSA) targets the minimization of the maximum difference between desired and actual throughput, across all active traffic flows. The framework was integrated into an O-RAN platform, and is deployed as an xApp in the Near-RT RIC. The deployed framework is very flexible, and can adapt its architecture based on the number of traffic flows. Additionally, it is possible to create multiple policy instances, each independently and sequentially serving a subset of users, improving the framework’s scalability.

The research conducted in [47] employs RL for adaptive resource allocation, demonstrating its utility in the context of Non-RT RIC. The ML agent deployed in the Non-RT RIC acquires knowledge and learns a radio resource allocation policy capable of adapting to dynamic environments, while simultaneously meeting diverse energy-driven criteria. Within the learning context, key information such as the mean and variance of the channel quality indicator (CQI) from the previous period and the bit number of new data are aggregated at the onset of each period. Subsequently, three transmit parameters, including transmit power, the highest MCS, and the maximum transmission airtime, are selected and transmitted to the Near-RT RIC so that the transmit parameters can be applied to BSs. The chosen resource allocation policy’s effectiveness is evaluated at the end of each period by the Near-RT RIC, which computes a reward indicative of the transmission rate. This reward is then sent to the Non-RT RIC. Through this iterative learning process, the ML agent gradually acquires knowledge and discerns the optimal resource allocation policy capable of adapting to dynamically changing environments.

**Table 7:** Summary of current efforts on ML applications for O-RAN resource management.

Year	Ref	Contribution
2022	[44]	Demonstrated the performance of on-policy and off-policy DRL methods in the form of PPO and ACER, respectively, and the performance was compared in an O-RAN setup for resource allocation optimization in a real-time surveillance video application.
2022	[45]	Employed a policy gradient-based RL algorithm as an alternative to the initially proposed ILP, to solve an MDP and address the challenge of fairly allocating limited resources to eMBB and URLLC users from multiple O-RUs while providing a significantly less complex solution.
2021	[46]	Proposed a RL-based dynamic resource controller leveraging policy differential semi-gradient SARSA to optimize traffic flow management by effectively and dynamically allocating per-flow resources within an O-RAN platform.
2021	[47]	Designed ML deployed in a Non-RT RIC to adapt the resource allocation policy to environment dynamics while satisfying various energy-driven criteria.

### 3.6. Session Management

Session management in O-RAN refers to the process of establishing, maintaining, and terminating communication sessions between network components. These sessions are used to transmit data, control signals, and other information between different components of the network. Session management is an essential function of O-RAN, as it enables the coordination and control of network operations and services. It involves the management of session parameters, such as session identifiers, session timeouts, and session initiation and termination procedures. Effective session management in O-RAN is critical for ensuring the efficient and reliable operation of the network, as well as for enabling the delivery of high-quality services to end-users.

The authors of [48] explore the efficiency of RL-based methods for intelligent session management when taking advantage of the intelligent gNB architecture of O-RAN. This architecture is again assumed to facilitate the inclusion of AI/ML algorithms mainly due to the introduction of the RIC, which is designed for sustainable deployment of these algorithms. The work is focused on the lack of effort to reduce the packet transmission latency in the Core and Data Networks, which can go up to hundreds of seconds compared to several milliseconds in the gNB fronthaul/backhaul links. By installing intelligent session management schemes based on policy evaluation RL methods such as Q-learning, double and State-Action-Reward-State-Action (SARSA) on the RIC of an O-RAN emulated gNB, it becomes able to effectively predict room to accommodate new PDU sessions with given service requirements. Therefore, the gNB can decide whether to grant a new PDU session or QoS flow, preventing existing and new sessions from violating the latency requirements.

In [49], the authors study connection management under the session management function (SMF), specifically for user-cells associating the user with a BS, considering sub-optimal and greedy solutions such as the received signal reference power (RSRP). Even though the greedy approach is simple and effective, it does not take into consideration the network’s local and global status. This causes the lack of load balancing where a certain BS can be overloaded while the neighboring BSs are underutilized. So, the O-RAN architecture features support global RAN automation to balance the load over the network resources by deploying ML algorithms as rApps and xApps in the RIC. The authors proposed a Deep Q-learning algorithm to infer the weights of the graph neural networks (GNN) for optimal user-cell association.

While user access management in O-RAN refers to the process of controlling and securing access to network resources and services by users and applications, it includes the management of user identities, authentication, authorization, and accounting (AAA). The goal of user access management is to ensure that only authorized users and applications can access the network, and that they can do so in a secure and controlled manner. User access management in O-RAN typically involves the use of access control policies, authentication mechanisms, and auditing tools to manage user access and monitor network activity. It is a critical component of network security and privacy, as unauthorized access to network resources can lead to data breaches, network disruptions, and other security incidents. Effective user access management is essential for ensuring the secure and reliable operation of O-RAN with session management.

In [50], the authors address the anticipated handover-rate and load balancing issues if O-RAN is deployed under the conventional user access control schemes typically based on signal strength or capacity measurements. This is a result of the openness nature of O-RAN, where the BS functions are decomposed and virtualized into CUs, DUs, and RUs, where they are massively deployed throughout the network. Therefore, typical access control procedures would make this a practically intractable process due to the massive signaling overhead and system complexity. This can be considerably mitigated if each UE autonomously selects proper BSs (or CUs/DUs/RUs). In this direction, a federated DRL-based scheme to address user access control in the O-RAN is proposed to establish intelligent user-centric access control mechanism to optimize the overall throughput and avoid frequent handovers. This is achieved by enabling the UE to train two deep Q-networks (DQNs) using its own observations, and making access decisions based on its DQNs outputs. Then, the DQN parameters are forwarded to a global model server installed in the RIC. This server can select just a group of UEs in each instance to further mitigate the signaling overhead. Afterward, the global model is updated by aggregating DQN parameters obtained from the selected UEs. The DQN global parameters are then disseminated to each UE to further improve its access decision. The achieved results show that the proposed scheme can reduce the frequency of handover in a O-RAN environment up to 53% when compared to a conventional access control based on signal strength UE measurements.

**Table 8:** Summary of current efforts on ML applications for O-RAN session management.

Year	Ref	Contribution
2021	[48]	Applied an ML algorithm in O-RAN to maintain QoS satisfaction by controlling admission of PDU sessions.
2021	[49]	Applied DQN to optimize user-cell association in O-RAN by supporting the global RAN automation through load balancing over the network resources.
2021	[50]	Proposed a smart user-centric access control in O-RAN using Federated DRL-based learning to mitigate frequent handover.

### 3.7. Traffic Steering

O-RAN traffic steering research aims to optimize network traffic management by intelligently routing traffic based on real-time network conditions and user demand. This involves analyzing various network parameters, such as traffic load, network congestion, and user behavior, to determine the best path for traffic flow. Traffic steering techniques can also be used to dynamically split traffic across different network functions and resources in real-time, improving network performance and reducing latency. ML algorithms can play a critical role in traffic steering research, providing real-time intelligence and decision-making capabilities to optimize traffic flow and improve overall network performance. However, there are still challenges related to scalability, complexity, and limited resources that need to be addressed to achieve optimal traffic steering in O-RAN.

In O-RAN, more processing is done by placing virtual network functions (VNF) in the DU before transferring data over midhaul links. This means that placing VNFs in the CU needs more bandwidth compared to placing in the DU [60]. Therefore, the question is how many functions should be left in the DUs to prevent network congestion on midhaul links arises. This is especially the case when DUs have limited computing/storage capacities compared to CUs. The authors of [61] have proposed an optimization problem to select the split points in O-RAN. The objective of this study is to balance the load across CUs as well as midhaul links with respect to the required delay and bandwidth and processing capacity of the DUs and CUs. Going beyond the static optimization, it is also noted that in real-world scenarios under traffic demands dynamicity and uncertainty at RUs, methods like RL and DRL can be used to provide dynamic VNF splitting across CUs and DUs.

In [62], O-RAN decouples the Control Plane (CP) from the User Plane (UP) through the E1 interface, which is derived from the software defined network (SDN) architecture. This separation of CP and UP allows a network to be more flexible in programming. The CP is implemented in hierarchical RICs, and manages radio resource functions through A1 and E2 interfaces. The authors of [62] proposed using the hierarchical RICs to minimizes end-to-end delay of the data plane traffic by placing Containerized Network Functions (CNFs) effectively. In comparison to VNFs, CNFs are lighter and can be implemented through microservice architectures, enabling a dynamic, scalable, and flexible architecture towards 5G [63].

Additionally, paper [4] discusses the use of ML methods to achieve modular and flexible O-RAN implementations in 6G networks, with a focus on the traffic steering use case and O-RAN xApps. The authors describe several ML algorithms that can be used for traffic steering, including decision trees, k-nearest neighbor (KNN), and neural networks. They also discuss the use of RL to train an agent to make traffic steering decisions in real-time. In [51], a federated meta-learning approach for traffic steering in O-RAN systems is proposed. This approach allows multiple Radio Access Technologies (RATs) to learn from each other without sharing their private data. The authors present a neural network architecture that uses meta-learning to adapt to different RATs and learn to steer traffic in a decentralized manner.

Paper [52] proposes a traffic steering use-case in O-RAN systems that exploits the benefits of Non-Orthogonal Multiple Access (NOMA) to improve radio resource efficiency. The authors introduce a resource allocation algorithm that dynamically allocates radio resources based on the traffic demand and channel conditions of the users. The proposed algorithm leverages NOMA to allow multiple users to share the same radio resources, while ensuring a high-quality user experience. Paper [64] proposes a traffic steering approach that ensures the efficient coexistence of eMBB and uRLLC services in O-RAN systems. They introduce a multi-objective optimization problem and a traffic steering algorithm that dynamically steers traffic based on the network load and user demands while ensuring a minimum QoS requirement for both eMBB and uRLLC users.

**Table 9:** Summary of current efforts on ML applications for traffic steering in O-RAN.

Year	Ref	Contribution
2021	[4]	Proposed logistic regression for modular and flexible O-RAN in 6G networks, focusing on traffic steering and O-RAN xApps.
2022	[51]	Introduced federated meta-learning with DQN for privacy-preserving multi-RAT knowledge sharing.
2022	[52]	Proposed Q-learning-based algorithm for power and frequency allocation in O-RAN to minimize macro gNB interference and maximize device QoS.

### 3.8. Mobility Management

Mobility management is a key function for cellular communication to maintain service continuity and ensure a good level of service quality for users moving across a network. This requires efficient coordination of radio resources to achieve predictive, timely, and successful handovers for preventing communication disruptions in highly dynamic mobile environments. To support this, the O-RAN architecture offers various capabilities, including the collection of, maintenance of, and access to historical traffic and radio data. Additionally, real-time monitoring of traffic and radio conditions is achievable through the support of the Near-RT RIC framework, which enables the deployment of AI/ML-based applications for detecting and predicting handover anomalies at the user level.

In [53], the authors proposed a new predictive handover method to predict target cells in advance, hence to reduce handover failures. Handover cases are simulated by random user movement within an environment with three eNBs and coverage holes. The handover prediction algorithm is implemented within a software developed by O-RAN Software Community (O-RAN SC), where an Anomaly Detection use case has been installed in the Near-RT RIC platform, which consists of three xApps: Anomaly Detection, Traffic Steering, and QoE Predictor. The process starts with the Anomaly Detection xApp, which analyzes UE data and sends notifications to the Traffic Steering xApp via the RMR protocol when anomalies are detected. The Traffic Steering xApp then requests a prediction of the target cell from the QoE Predictor xApp, which uses the Vector Autoregressive (VAR) algorithm to forecast time-series data based on past throughput data. As user mobility is not considered by the original QoE Predictor xApp, the paper has contributed by adding mobile users’ RSRP measurements to the predict cells’ throughput. The proposed intelligent prediction method achieves higher successful transmission rates than conventional handover algorithms and allows the traffic steering xApp to send commands to the RAN, such as a handover command using the REST API interface.

**Table 10:** Summary of ML studies for mobility management in O-RAN.

Year	Ref	Contribution
2022	[53]	Implemented a NN-based handover prediction method for the next target cell in Near-RT RIC using software developed by O-RAN Software Community (O-RAN SC).

### 3.9. Energy Efficiency

Energy efficiency is a significant challenge in O-RAN due to their highly flexible and scalable design, where several components and technologies must work together seamlessly. Achieving energy efficiency also requires balancing trade-offs with other performance metrics such as latency or throughput. Although dynamic function splitting and ML-based optimization techniques can be used to allocate computing resources efficiently, challenges related to limited resources, scalability, and the dynamic environment of O-RAN must be addressed to achieve optimal energy efficiency.

To address this, the authors in [54] propose an online learning-based energy-aware scheduling method for virtualized Base Stations (vBS) in O-RAN. The goal is to optimize scheduling policies that reduce energy consumption while maximizing vBS performance. The novelty of this work lies in the application of adversarial bandit learning to vBS operations. The authors introduce a Policy Decider application within Non-RT RIC to learn and implement optimal policies, which can be adjusted based on network conditions and user needs. The policy decision is shared with Near-RT RIC through A1 interface, and the Data Monitor calculates the reward based on achieved performance and energy cost, which is then sent to the Policy Decider via the O1 interface at the end of each decision time slot. Data-driven experiments based on real-world traffic traces and testbed measurements are conducted to evaluate the effectiveness of the proposed method and compare it to state-of-the-art approaches. The proposed approach outperforms state-of-the-art methods by achieving energy savings between 35.5% and 74.3%, as demonstrated through data-driven experiments based on real-world traffic traces and testbed measurements.

**Table 11:** Summary of ML studies for energy efficiency in O-RAN.

Year	Ref	Contribution
2022	[54]	Proposed online adversarial bandit learning for energy-aware scheduling policy optimization to maximize the performance of virtualized Base Stations (vBS) in O-RAN.

### 3.10. Satellite NTN

Interference management in satellite networks involves reducing interference between multiple satellites and traditional cellular communication systems, which can lead to degraded signal quality and decreased network performance. O-RAN can be used for interference management in satellite networks by leveraging its cutting-edge functionalities. One way to use O-RAN for interference management in satellite networks is by using a dynamic spectrum access techniques to dynamically allocate and manage spectrum resources based on real-time network conditions. This involves monitoring network parameters, such as channel quality and interference levels, and dynamically reconfiguring the network to avoid interference and optimize network performance. Another approach is to use machine learning algorithms to analyze network data and make intelligent decisions on interference management. For example, machine learning algorithms can be trained to identify patterns in network data that indicate interference and automatically adjust network parameters to mitigate interference. Finally, O-RAN can also be used to implement beamforming techniques to improve signal quality and reduce interference in satellite networks. Beamforming involves adjusting the phase and amplitude of transmitted signals to create directional beams focused on specific network areas. By optimizing beamforming parameters based on network conditions, interference can be minimized and network performance improved. Additionally, the radio spectrum is a finite and highly sought-after resource; therefore, spectrum sharing aims to help resolve this issue by creating regulatory frameworks and developing wireless technologies to share spectrum bands between heterogeneous users. The authors in [55] proposed O-RAN with machine learning for 5G/XG and closed-loop feedback via sensing can reduce harmful interference between heterogeneous 5G and Low Earth Orbit (LEO) satellite communication systems.

**Table 12:** Existing study of ML applications for Satellite NTN relevant to O-RAN.

Year	Ref	Contribution
2021	[55]	Investigated how O-RAN can be used for 5G/XG to mitigate interference between terrestrial and space communication systems.

## 4. Research Opportunities in O-RAN

Applying ML in O-RAN networks has the potential to significantly improve network performance, automate complex network operations, and enable new use cases and business models. ML algorithms can analyze large volumes of network data, enabling intelligent decision-making in real-time and optimizing network resources for enhanced user experiences. In the previous section, we provided overview of the state-of-the-art research works that demonstrate the use of ML to improve O-RAN and the underlying network elements. However, challenges remain to apply and integrate ML into O-RAN for network automation. These challenges include the need for high-quality and diverse training datasets, ensuring robustness and fairness of ML models, addressing privacy concerns, and developing efficient computational frameworks capable of handling the scale and complexity of O-RAN deployments. In this section, we shall describe the potential of ML applications in O-RAN and their challenges. Table 13 shows a summary of identified research opportunities.

### 4.1. Proactive Maintenance

A significant fraction of the cost of maintaining a cellular radio network is due to the need for site visits. Virtualization of the RAN within the O-RAN framework may help considerably by extending the scope for remote or automatic intervention to mitigate hardware failures, but ultimately the only way to repair faulty on-site hardware is by making a site visit. Ideally, it would be possible to predict failures before they occur, allowing proactive scheduling of site visits. Deep learning systems can potentially assist here by trawling very large datasets to detect “precursor” events occurring before failures, which might be difficult to find by an engineer tasked with scanning the fault logs. Before this can happen, however, at least two open issues need to be addressed. The first is to evolve the O-RAN framework in conjunction with ML system design so that detailed fault-related data are provided at the O1 interface, including additional information required by the virtualization of the RAN. The second is related to the fact that in a reliable network fault data are relatively scarce, with the further issue that equipment from different vendors may operate in slightly different ways. Here, the challenge is to develop ML training approaches which can be applied in a standardized way across the network, irrespective of equipment vendor or site location.

In relation to the first issue, in [65], members of Chungwha Telecom Taiwan and their academic partner present a view of a proposed high level architecture for an O-RAN Network Management System from the network operator’s perspective. The authors describe the architectures for 3GPP NG- and O-RAN, and provide a list outlining the key Operations and Maintenance (OAM) functions supported by the O-RAN O1 interface. They then provide a high-level layered architecture for an O-RAN-based network management system, together with a diagram indicating the relationships between the proposed NMS and other system entities. The paper references a TM Forum white paper published in 2003 on the New Generation Operations System and Software (NGOSS), but at this stage no attempt is made to evaluate this in relation to the current O-RAN context, where extensive use is made of virtualized network functions. The authors suggest that further work is needed to clarify the work distribution between the network management system and the entities managing the relevant clouds, as well as the approach to management of the virtualized O-RAN elements.

On the second issue, one opportunity to increase the amount of available fault data could be to make use of the emergence of self-healing networks, especially in the context of virtualization, in which faults being compensated for by the self-healing process may persist in dormant form for some considerable time and yield valuable information.

Another possibility is suggested by Mulvey et al. [66], in which they survey the literature on fault management in cellular networks and outline a number of suggestions for further work which are relevant to O-RAN. In particular, they consider the issue of multi-vendor equipment configurations, and suggest that transfer learning may be a possible approach, especially for distributed ML systems, which would allow ML subsystems to be trained on one vendor’s equipment, and the learning approach transferred with appropriate adjustments to equipment provided by other vendors.

Recently, there has also been growing interest in federated learning [67], which can potentially harmonize data across multiple site locations, allowing a centralized ML system to utilize data from a large number of locations, when translated into a common format.

### 4.2. xApps, rApps and dApps Operation

The O-RAN architecture allows the RIC to host and run applications developed by third party for automation and intelligent orchestration to the network through ML and AI, which will leverage the enormous amount of data generated by the RAN and exposed through the O-RAN interfaces to analyze the current network conditions, forecast future traffic profiles and demand, and implement closed-loop network control strategies to optimize the RAN performance. The add-on xApps, rApps, and dApps will make the monolithic RAN “black-box” obsolete and provide open, programmable and virtualized solutions that expose status and offer control knobs through standardized interfaces [68]. The rApps are residence of the Non-RT RIC and control the optimization objectives such as policies, models and slicing that the time scale of the close control loop of the network requires more than one millisecond. The Near-RT RIC hosts the xApps that require the response of control loops time between 10 and 1000 ms for optimizing objectives such as the RRM and session management. However, the notion of dApps, custom and distributed applications can complement xApps/rApps by implementing RAN intelligence at the CUs/DUs for real-time use cases outside the timescales of the current RICs. The control loop response timescale in such use cases is ≤10 ms to optimize objects such as the beamforming and modulation management. The dApps receive real-time data and KPMs from the RUs (e.g., frequency-domain I/Q samples), DUs (e.g., buffer size, QoS levels), and CUs (e.g., mobility, radio link state), as well as enrichment information from the Near-RT RIC, and use it to execute real-time inference and control of lower-layer functionalities. Such dApps enable network intelligence at the edge of the O-RAN ecosystem [69].

It is clear that intelligent and dynamic xApps, rApps and dApps are key enablers for future network automation. However, it also introduces novel practical challenges concerning. One of the challenges is the orchestration of the existing xApps, rApps, and dApps in the O-RAN RIC. In addition, the question is how to maintain the orchestration cross domain with core network SON functions in 3GPP and any newly added xApps, rApps, and dApps to the RAN without creating conflicts between all these apps. As a result, the network intelligence orchestration for the different types such as the xApps, rApps, and dApps is an unprecedented problem that requires innovative, automated and scalable solutions. In [69], the authors formulate an orchestration problem where the orchestration policy variable *X* is computed to maximize the total value of requests being accommodated by selecting (i) which requests can be accommodated; (ii) which AI/ML models should be instantiated; and (iii) at what location the AI/ML and requests should be executed to satisfy request performance and timescale requirements to avoid or mitigate the conflict between then and at the same time, complying with the requirements of each request [70].

### 4.3. Satellite NTN

One of the challenges of Satellite NTN low-latency communications is long latency due to the significant distance between the terrestrial UE and the satellite [71,72,73]. Using a distributed computing model, O-RAN can help a satellite solve its latency drawback. In this model, the O-RAN intelligent controller can be deployed closer to the end-user (for example, at the satellite gateway or user terminal) to reduce the round-trip time for control and management signals. The controller can also use advanced algorithms to intelligently allocate radio resources, reducing the need for frequent signaling between the user terminal and the satellite. Another way O-RAN can help reduce latency is through the use of edge computing. Edge computing involves moving tasks closer to the user or device, reducing the amount of data that must be sent back and forth between the user and the satellite. This approach can be used to run applications such as video streaming, gaming, or IoT applications, which require low-latency and high-bandwidth connections.

For massive devices involved in NTN, ML-empowered O-RAN architecture can play an essential role in optimizing the performance, e.g., throughput, coverage probability, latency, and energy efficiency. Firstly, for capacity optimization, ML can help predict the capacity requirements of the NTN and optimize the allocation of resources such as bandwidth, power, and antenna coverage. Secondly, ML can help identify and mitigate network interference sources, such as adjacent satellite interference, co-channel interference, and inter-system interference. Thirdly, ML can optimize the energy consumption of the O-RAN-assisted NTN, such as by reducing the power consumption of individual network elements or adjusting the power levels based on traffic demand. Finally, as latency remains one of the main challenges in Satellite NTN, reducing latency is important for Satellite NTN to support a wide range of applications. Some recent papers introduce the use of generative AI and digital twin in the communication system to deal with bandwidth limitation and, particularly, the latency [74]. As O-RAN offers convenient hosting of ML models, ML models can be pervasively deployed in O-RAN to encourage use of generative AI and digital twin in satellite communications.

### 4.4. Massive MIMO

In the last two decades, the idea of using multiple antennas for transmitting and receiving information over the air has evolved from the classic single-cell MIMO technology, to the distributed cooperative massive MIMO with no cell boundaries technology, also known as cell-free technology. With mMIMO and cell-free becoming ubiquitous in 5G and 6G, O-RAN will need to accommodate these technologies and provide the right interfacing for making the most of their large spectral efficiency (SE) potential. In this regard, the work of [75] looks at how mMIMO and cell-free can be integrated in O-RAN. They provide several options of integrating the multi-antenna processing (mMIMO precoding/beamforming) of cell-free MIMO in the O-RAN architecture, by adjusting the level of coordination/cooperation between the open-distributed units (ODUs) and open-radio units for performing this processing. It turns out that increasing the centralization (exchange of information between the various units) increases significantly the SE. Even though the current O-RAN architecture can, in their view, already support cell-free networks, there are opportunities for achieving higher SE in the future by specifying the inter-O-DU interface in O-RAN and performing the multi-antenna processing at the O-DU. Finally, they also point out the importance of RU clustering (which is, itself, tied to user grouping) for achieving better SE performance; this could be efficiently implemented at the Near-RT RIC in O-RAN. We can foresee that AI and ML will help to dynamically adjust the level of coordination/cooperation between the ODUs, as well as efficiently perform the RU clustering.

As pointed out by [75], the integration of the multi-antenna processing for the mMIMO/cell-free technology is clearly an important issue in O-RAN, given the split of baseband functionalities, and the work [76] investigates this issue in a more practical manner. More specifically, it investigates how to effectively distribute the channel state information (CSI) between the split baseband functions to minimize the performance degradation it incurs when performing multi-antenna processing. This work also identifies further research opportunities on the same topic, as for instance, the optimization of the fronthaul bandwidth allocated to different users according to their mobility, priority, or channel conditions, and reduce the exchange of information over the fronthaul interface between O-DU and O-RU. This optimization process can obviously made more generic and efficient by using ML.

Another important technology that will deploy in 6G is the intelligent reflective surface (RIS) technology. The idea behind RIS is to increase the SE/EE of wireless communications systems by dynamically improving the propagation environment, without the need of deploying extra costly access points (APs). The work in [77] mentions the possible integration of RISs in the O-RAN architecture, where RISs are managed via a dedicated controller. This controller is then linked to O-RAN via a new interface at the Near-RT RIC. Accordingly, it is clear that the design of a practical and effective signaling interface will be the main challenge for integrating RISs into the O-RAN architecture. This work sees the deployment of RISs as an the opportunity to create fully inter-operable so-called ’smart-radio environments’ which, in turn, can provides more openness and flexibility for the network operators. The management of such ’smart-radio environments’ will require intelligence provided by AI/ML.

### 4.5. Mobility Management

In V2X communication, the meticulous design of an efficient handover strategy holds paramount importance in effectively managing challenges such as short stays, ping-pong effects, and remote cell scenarios. Furthermore, with the proliferation of UAVs across various applications, such as agricultural plant protection, police enforcement, and environmental monitoring, the reliability of connection, inherently influenced by mobility factors, emerges as a critical area of investigation.

Within the framework of the O-RAN architecture, ML emerges as a powerful tool for designing a proactive and data-driven strategy for mobility management. Specifically, leveraging Non-RT RIC, multi-dimensional data can be obtained, including metrics derived from vehicle-related measurements based on UE reports, trajectory information pertaining to vehicle paths, and spatial constraints. Subsequently, using this acquired data, the Non-RT RIC can construct machine learning models by leveraging historical information, enabling informed decisions to facilitate reliable connection support. These ML models, once constructed within the Non-RT RIC, can be effectively deployed and executed by the Near-RT RIC, enabling it to discern optimal radio resource configurations for establishing and maintaining dependable communication links [78].

As highlighted in [79], the integration of flying UAV BSs with O-RAN introduces notable challenges concerning agility, distributed computation, and dynamic mobility of UAVs. The efficient control of UAV-BSs can be significantly enhanced through the utilization of intelligent O-RAN functionalities, playing a pivotal role in addressing the requirements of unforeseen applications where terrestrial networks may prove inadequate. In this context, it would be imperative to explore innovative approaches rooted in ML for jointly optimizing the trajectory of UAVs acting as flying BSs and the task offloading among the diverse O-RAN elements, including O-RU, O-DU, and O-CU. The comprehensive evaluation of performance metrics, encompassing resource utilization, service acceptance rate, and utility values, alongside a multi-agent learning framework, becomes essential [79].

In mobility management, location information provides an additional dimension of inputs to improve its decision making. Localization techniques can also be enhanced using ML techniques [80]. With O-RAN, not only can ML techniques can be introduced into the network to enhance the localization, but the output of localization can, in turn, provide useful inputs to improve the mobility management in O-RAN.

### 4.6. Network Management

As mobile networks become increasingly complex, there is a growing need for advanced solutions to effectively manage network operations. Although research has explored the application of ML techniques for automating network management, further efforts are required to enhance various aspects of network management. O-RAN, with its open architecture, presents a convenient platform for leveraging ML techniques in network management. The flexibility and openness of O-RAN enable the seamless integration of ML-based approaches, providing opportunities to enhance and optimize various aspects of network management.

For supporting different network slices (e.g., eMBB, URLLC, and mMTC slices), the efficient placement of VNFs of slices onto the network infrastructure is crucial. The necessity of the optimization of the functional split of individual RAN slices between CU, DU, and RU entities, based on the functional split options defined by 3GPP, is studied in [81]. In addition, the optimal placement of RAN slices in a multi-tier 5G Open RAN architecture, including multi-tier aggregation sites, has been emphasized in [81] by demonstrating that a flexible functional split can lead to enhanced utilization of physical network resources. Considering the various types of data available, such as network traffic patterns, resource usage availability, and future resource demand, these can be leveraged to determine the VNF split for each network slice. By harnessing the power of ML, characterized by its remarkable ability to forecast future patterns and make data-driven decisions, the flexible function split can be dynamically adjusted in response to evolving network environments, thereby optimizing target objectives encompassing resource utilization, data rate, power efficiency, and cost-effectiveness.

### 4.7. Data Privacy and Security

As ML relies heavily on data, ensuring the privacy and security of sensitive network data are paramount. O-RAN handles vast amount of data, including user information and network configurations. It is crucial to manage ML algorithms to access and utilize these data without compromising user privacy. In addition, O-RAN often involves in collaborations between different operators and vendors. Then, secure data sharing protocols must be established to ensure that sensitive network information is shared only with authorized parties. ML models requiring data from multiple sources should adhere to strict data sharing policies. As O-RAN becomes more software-centric and dynamic, it can be vulnerable to cyberattacks. While ML can be used to detect and respond to threats, it is crucial to secure ML models themselves against adversarial attacks [16]. In this case, what measures can be implemented to protect against potential data breaches or cyberattacks targeting ML models is worth investigating. Striking the right balance between data accessibility for ML and maintaining robust data security would be critical for network operators and developers. Successfully addressing the data privacy and security challenge will be essential to foster trust in ML applications with O-RAN and ensure compliance with evolving data protection regulations.

### 4.8. Big Data Collection for Machine Learning

Given its openness, using O-RAN for big data collection in the context of ML offers substantial advantages. O-RAN promotes interoperability among different vendors’ network components, allowing for diverse data sources, which is vital for ML model training and accuracy. It also fosters vendor neutrality, reducing lock-in and enabling network operators to select the best-suited hardware and software components, enhancing data collection capabilities. The scalability of O-RAN accommodates the growing volume of data generated by modern networks and encourages innovation by enabling custom software applications, including data collection and analytic tools tailored to specific ML use cases. O-RAN development can further advance with the more data available for ML, including use of synthetic data from network simulators [82]. Simulation tools such as OpenRAN Gym [83] and WiThRay [84] offer data generation for ML training when empirical data are insufficient or difficult to collect. However, data may be out-of-distribution due to different domains, and new learning techniques should be explored to tackle the out-of-distribution issue [85].

## 5. Conclusions

ML applications have sparked significant interest in O-RAN for their potential to revolutionize network automation. By exploiting ML to analyze vast amounts of network data in real-time, identifying performance issues and optimizing network parameters on the fly, ML is expected to facilitate predictive maintenance, intelligent resource allocation, and network optimization. Furthermore, predictive analytics can help anticipate and prevent network failures, reducing downtime and maintenance costs. Ultimately, ML holds the promise of making O-RAN networks more efficient, reliable, and responsive to the dynamic demands of modern communication environments.

In this paper, focusing on network automation in O-RAN using ML techniques, we first presented the design principles and architecture of O-RAN, highlighted the openness and disaggregation of RAN components, and its capability to extend network operation with native ML support. The current research landscape of ML applications in O-RAN was then presented. Several key aspects of network management were surveyed, including session and user access management, radio resource management, network slicing, mobility and traffic management, energy efficiency, O-RAN component deployment and function splits, ML workflow management, and support for NTN and satellite networks. For instance, ML can play a pivotal role in automating fault detection and recovery processes by meticulously analyzing data traffic and identifying anomalies. This reduces the necessity for manual intervention, subsequently bolstering network resilience. ML can also prove invaluable in the realms of capacity planning and elevating customer experiences. By forecasting network traffic growth, aiding in optimizing capacity, and analyzing user behavior and feedback, ML can contribute to a more efficient and customer-centric network management approach.

However, numerous challenges must be surmounted to unlock the full potential of ML in propelling O-RAN network forward. Upon identifying several pivotal research domains, these challenges manifest across diverse realms encompassing data collection and analysis, as well as the development, deployment, maintenance, and operation of ML models. A fundamental hurdle arises from the various types of data emanating from different vendor equipment, necessitating harmonization for effective data analysis. Utilization of synthetic data collectible from network simulators could be also considered when real-world empirical data are difficult to obtain. Once data acquisition hurdles are overcome, data-driven decision-making processes must be thoughtfully tailored for target optimizations, such as enhancing network capacity and mitigating interference. In complex scenarios (e.g., O-RAN-including UAVs), consideration of multiple objectives would be required simultaneously, such as optimizing the trajectory of UAVs and task offloading among heterogeneous O-RAN components, including UAVs. The multifaceted nature of O-RAN components provide various options for deploying ML models, prompting careful deliberation on compatible components and interface for ML model and data exchange. When multiple components are selected for ML model deployment, the level of coordination and cooperation between components should be also investigated. Furthermore, the dependable operation of multiple ML modules such as xApps, rApps, and dApps within O-RAN RIC, mandates unwavering attention to reliability. Equally critical is the dynamic and autonomous responsiveness of deployed ML models to ever-changing network environments. Furthermore, we discussed the issue to find the balance between data accessibility for ML and maintaining robust data security.

These challenges, when conquered, will usher in an era where ML empowers O-RAN network automation, leading to heightened efficiency, reduced operational costs, fortified security, and an enriched user experience. They also position network operators to adapt to the evolving demands of the communication landscape, conferring a competitive edge in this rapidly transforming terrain.

## Figures and Tables

**Table 1:** Summary of surveys relevant to ML-enabled O-RAN. H: High, M: Medium, and L: Low.

Year	Ref	O-RAN Architecture	ML Application in O-RAN	Research Opportunities	Remarks
2023	[1]	H	M	M	This paper provides a detailed tutorial on O-RAN, describing its architecture, design principles, and the O-RAN interfaces.
2021	[4]	H	L	L	This paper overviews the idea of O-RAN and presents ongoing O-RAN Alliance standardization activities in this context, followed by a study of traffic steering use case.
2022	[12]	H	L	L	This paper provides a comprehensive survey of O-RAN development, encompassing a summary of the RAN evolution history, an introduction to O-RAN technology, an overview of Open RAN-related projects and activities, a discussion of standardization efforts, challenges, and potential solutions.
2021	[13]	M	L	L	This paper provides an overview of the O-RAN Alliance RAN architecture, highlighting its core building blocks and, subsequently, it presents a practical use case that leverages AI/ML-based innovations.
2022	[14]	H	M	M	This paper presents an O-RAN architecture overview, delves into AI applications within Op-RAN, and discusses the challenges and opportunities in implementing intelligent solutions in 5G and B5G telecommunications.
2022	[15]	H	M	M	This paper focuses on mapping existing deep-learning-based studies to the O-RAN architecture, highlighting key technical challenges, open issues, and future AI-enabled O-RAN research directions.
2023	[16]	M	L	L	This paper examines security and privacy risks in O-RAN architecture, proposes possible solutions and presents relevant security standardization efforts.
2023	[17]	M	L	L	This paper focuses on Non-Terrestrial Networks exploring the possible implementation of an O-RAN-based NTN solution.
2022	[18]	H	L	L	This paper identifies critical limitations in current O-RAN specifications: security, latency, real-time control, and AI-based RAN control.
2023	[19]	H	M	L	This paper focuses on XAI methods and explores their deployment within the context of O-RAN.

**Table 2:** The structure of Section 3 for ML applications in Open RAN.

Title	Ref	Title	Ref
Section 3.1 O-RAN Deployment	[15,35]	Section 3.2 AI/ML Implementation	[22,36]
Section 3.3 Network Slicing	[37,38,39,40]	Section 3.4 Dynamic Function Split	[41,42,43]
Section 3.5 Resource Management	[44,45,46,47]	Section 3.6 Session Management	[48,49,50]
Section 3.7 Traffic Steering	[4,51,52]	Section 3.8 Mobility Management	[53]
Section 3.9 Energy Efficiency	[54]	Section 3.10 Satellite NTN	[55]

**Table 13:** Research opportunities for ML applications in O-RAN.

	Category	Issues
Section 4.1	Proactive Maintenance	- How to evolve the O-RAN framework in conjunction with ML system design; - To develop ML training approaches across the network irrespective of equipment vendor or site location (i.e., different configurations of multiple vendor equipment and unharmonized data across multiple sites).
Section 4.2	xApps, rApps, dApps Operation	- Orchestration of xApps, rApps, and dApps in the O-RAN RIC when they are simultaneously operated for network automation; - Orchestration across the domain with SON functions in a core network and any newly added xApps, rApps, and dApps to the RAN.
Section 4.3	Satellite NTN	- To optimize the resource allocation for capacity enhancement; - To mitigate network interference sources such as adjacent satellite interference and inter-system interference.
Section 4.4	Massive MIMO	- To provide right interfaces to integrate multi-antenna processing in O-RAN to maximize the spectral efficiency; - To dynamically adjust the level of coordination/cooperation between DUs and to efficiently perform the RU clustering; - To effectively distribute the channel state information between the split baseband functions.
Section 4.5	Mobility Management	- To jointly optimize the trajectory of UAV and the task offloading among diverse O-RAN elements.
Section 4.6	Network Management	- To optimize the flexible functional split of RAN slices dynamically to respond to changing network environments.
Section 4.7	Data Privacy Security	- To make ML models to access and utilize the data without compromising user privacy; - To secure ML models against adversarial attacks and to develop the measure indicating protection against potential data breaches or cyberattacks.
Section 4.8	Big Data Collection for ML	- To improve data collection by using O-RAN, including collection and consolidation of hybrid empirical and synthetic data.

## Data Availability

No new data were created or analyzed in this study. Data sharing is not applicable to this article.
